# Human Fear Conditioning and Extinction in Neuroimaging: A Systematic Review

**DOI:** 10.1371/journal.pone.0005865

**Published:** 2009-06-10

**Authors:** Christina Sehlmeyer, Sonja Schöning, Pienie Zwitserlood, Bettina Pfleiderer, Tilo Kircher, Volker Arolt, Carsten Konrad

**Affiliations:** 1 Department of Psychiatry, University of Muenster, Muenster, Germany; 2 Interdisciplinary Center for Clinical Research (IZKF), University of Muenster, Muenster, Germany; 3 Department of Psychology, University of Muenster, Muenster, Germany; 4 Department of Clinical Radiology, University of Muenster, Muenster, Germany; 5 Department of Psychiatry und Psychotherapy, Philipps-University Marburg, Marburg, Germany; University of Nebraska, United States of America

## Abstract

Fear conditioning and extinction are basic forms of associative learning that have gained considerable clinical relevance in enhancing our understanding of anxiety disorders and facilitating their treatment. Modern neuroimaging techniques have significantly aided the identification of anatomical structures and networks involved in fear conditioning. On closer inspection, there is considerable variation in methodology and results between studies. This systematic review provides an overview of the current neuroimaging literature on fear conditioning and extinction on healthy subjects, taking into account methodological issues such as the conditioning paradigm.

A Pubmed search, as of December 2008, was performed and supplemented by manual searches of bibliographies of key articles. Two independent reviewers made the final study selection and data extraction. A total of 46 studies on cued fear conditioning and/or extinction on healthy volunteers using positron emission tomography or functional magnetic resonance imaging were reviewed. The influence of specific experimental factors, such as contingency and timing parameters, assessment of conditioned responses, and characteristics of conditioned and unconditioned stimuli, on cerebral activation patterns was examined. Results were summarized descriptively. A network consisting of fear-related brain areas, such as amygdala, insula, and anterior cingulate cortex, is activated independently of design parameters. However, some neuroimaging studies do not report these findings in the presence of methodological heterogeneities. Furthermore, other brain areas are differentially activated, depending on specific design parameters. These include stronger hippocampal activation in trace conditioning and tactile stimulation. Furthermore, tactile unconditioned stimuli enhance activation of pain related, motor, and somatosensory areas.

Differences concerning experimental factors may partly explain the variance between neuroimaging investigations on human fear conditioning and extinction and should, therefore, be taken into serious consideration in the planning and the interpretation of research projects.

## Introduction

Fear conditioning is an ability that is vital for the detection of danger, initiation of self-protection mechanisms, and for survival of a species. Disorders in humans associated with increased anxiety and fear levels, such as posttraumatic stress disorder, phobias, or panic disorder, exemplify how misguided fear conditioning might render originally innocuous stimuli fear-inducing and threatening. In addition, extinction of these associations is also hampered in these disorders. A life time prevalence of anxiety disorders of about 16,6% [1] highlights the substantial clinical and socioeconomic relevance of fear conditioning and extinction.

The term *conditioning* refers to the process of learning the association between two previously unrelated stimuli [2]. In a typical differential fear conditioning design, a previously neutral conditioned stimulus (CS+) is associated with an aversive and fear-inducing unconditioned stimulus (US) and becomes intrinsically aversive, while another neutral stimulus remains unpaired (CS-) [3]. Two main types of conditioning designs can be distinguished, which differ in the temporal relationship between CS+ and US, hence in the temporal *contiguity*. In *trace conditioning*, a time interval ranging from for example 500 milliseconds [4] to 10 seconds [5] separates the presentation of the CS+ from presentation of the US. The expression “trace conditioning” stems from the idea that a memory trace needs to bridge the gap between CS+ and the delayed US to form an association, therefore working-memory processes are more strongly involved in trace conditioning. In contrast, in *delay conditioning* the CS+ overlaps or is immediately followed by the US. A repeated exposure of the originally neutral stimulus without presenting the aversive stimulus gradually eliminates the fear reaction and is defined as *extinction*. In the past, extinction was regarded as a process of forgetting this association. However, the phenomena of *spontaneous recovery*, *renewal*, *rapid acquisition*, and *reinstatement* after extinction, suggest that fear extinction is “an active learning process that is distinct from acquisition and requires additional training to develop” [6].

Fear conditioning has proven to be an extremely robust, rapid, and precise experimental approach for studying the neurobiological substrates of fear [2], [7]–[15], while fear extinction most probably represents the main therapeutic ingredient of exposure-based psychotherapies. Numerous studies have investigated fear conditioning and extinction in animals and humans, resulting in a core neural network involved in conditioning and extinction (see e.g. [16]–[18]).

While the literature on animals has been summarized in several review articles (see e.g. [3], [6]), there has been no such approach in the current functional neuroimaging literature on human fear conditioning. So far, only a few reviews have been published and they focus on special topics such as extinction of conditioned fear [19], [20], or socio-cultural and cognitive influences on learning [21]. Büchel et al.'s (2000) review compared event-related fear conditioning studies to block-design studies and positron emission tomography (PET) studies. This important review was one of the first to identify a common core network for human aversive conditioning, including the amygdala and anterior cingulate cortex (ACC) [22]. Other reviews concentrated on cellular and synaptic mechanisms, or on plasticity within this neuroanatomical circuitry [3], [23].

Even though a core network for fear conditioning has consistently been reported in most imaging studies, results obtained from modern neuroimaging techniques differ in many respects, for example, in the number or the type of activated areas.

Therefore, the main aim of this review is to identify consistent and common findings on aversive conditioning and extinction in humans, as assessed by PET and functional magnetic resonance imaging (fMRI), and to present them in a structured manner. The second aim is to look at the differences between neuroimaging studies with respect to neuroimaging results and design parameters. We therefore identify and evaluate typical experimental factors that may influence brain activation patterns and may thereby contribute to the heterogeneity of neuroimaging results. Overall, this review is intended to facilitate the interpretation of seemingly contradictory neuroimaging findings, as well as the selection of an appropriate conditioning design for specific research purposes. Therefore, this review is relevant both to clinicians seeking for a state-of-the-art overview and to researchers investigating fear conditioning or extinction by means of neuroimaging.

The main results of the reviewed studies will be briefly summarized first, followed by an evaluation of specific consequences on activation patterns of critical factors concerning conditioning paradigms, measures of conditioning success, stimuli, and their timing. The review concludes with a critical discussion of these factors and an evaluation of their impact on past and future research.

## Methods

### Literature Search

To identify relevant neuroimaging studies on human fear conditioning and extinction, a computerized database search of journal articles via Pubmed was conducted for the years 1994–2008. This Pubmed search, as of December 2008, used combinations of the keywords “conditioning”, “extinction”, “aversive”, “fear”, “fMRI”, “neuroimaging”, “PET” and “humans”. No truncations and language restrictions were applied. We screened the abstracts for relevant literature based on the literature search criteria and additionally examined the references sections of articles and reviews for potentially useful studies.

### Selection criteria

Studies were included if they were: (1) PET or fMRI studies, (2) performed on healthy volunteers, (3) focused on cued fear conditioning and/or extinction. Furthermore, exclusion criteria were: (1) pharmacological modulation, (2) subliminal or masked presentation, (3) context conditioning, (4) combination of fear conditioning with other experimental tasks, such as cognitive-demanding working-memory tasks. Inclusion criteria were applied independently by two reviewers. Specific experimental designs for fear conditioning in fMRI and PET were compared, focusing on the impact of critical experimental variables, such as timing parameters, the contingency rate, or characteristics of the stimuli, on neuroimaging results.

### Data Extraction

Data were extracted by the first author (CS) and double-checked independently by the second author (SS). The discrepancies were resolved by consensus and the senior author (CK) was consulted if needed. The following variables were extracted and presented in Table 1: 1) demographic characteristics (number of participants, gender, and age), 2) study design (delay, trace, and extinction), 3) neuroimaging technique (fMRI, PET), 4) characteristics of the stimuli (modality of CS and US), 5) independent assessments of the conditioning process (e.g. heart rate), and 6) neuroimaging results. In the data analysis, the outcomes of interest were brain areas activated during conditioning and extinction. Therefore, we extracted the neuroimaging data presented by each study as the main results. Finally, we extracted those contrasts of interest that represent the conditioning or extinction effect (e.g. CS+>CS-).

**Table 1:** Forty-six studies on aversive conditioning and/or extinction, with forty studies on delay conditioning (including seven studies on extinction), two studies on trace, and four studies on delay and trace conditioning, with focus on main results of acquisition and/or extinction of conditioned responses (in alphabetic order).

No.	Study name	Subjects			Design	Technique	CS-US-contingency	CS	US	Independent assessment of the conditioning process	Neuroanatomical correlates of acquisition and extinction of conditioned responses
		N	M/F	Mean Age in years							
1	Anders et al. 2005	10	6/4	40	Delay	fMRI	50%	Neutral faces	Verbal	Online: SCR, startle eye blink amplitude, verbal reports of arousal and emotional valence	**Delay conditioning (assessed as (Acquisition > Habituation)):** MPFC (R), FOP (R)
2	Blaxton et al. 1996	7	1/6	27	Delay	PET	100%	Tones	Air blast	Online: eye blink	**Delay conditioning (assessed as (conditioning > pseudoconditioning)):** ACC, cerebellum (L, R), frontal L (L, R), hippocampal formation (R), lingual G (L), pons, thalamus (L).
3	Büchel et al. 1998	9	7/2	-	Delay	fMRI	50%	Neutral faces	Sound	Online: SCR	**Delay conditioning (assessed as (CS+unpaired>CS-)):** ACC (L, R), amygdala (L, R), ant insula (L, R), med parietal C (R), PMA (L, R), red N (L, R), SMA (R)
4	Büchel et al. 1999	11	6/5	-	Trace	fMRI	50%	Tones	Sound	Online: SCR	**Trace conditioning (assessed as (CS+unpaired>CS-)):** ACC (L, R), amygdala (L, R), post secondary auditory C (L, R), DLPFC (L, R), hippocampus (L, R), ant insula (L, R), vent putamen (L, R), med thalamus (L, R)
5	Carlsson et al. 2006	9	4/5	25	Delay	fMRI	100%	Visual Cue	Shock	Post: valence, pain- intensity, anxiety ratings	**Delay Conditioning (assessed as (correlated > uncorrelated trials):** med frontal L (R), post insula (L, R), SII (L, R), SI (L), hippocampus (L, R), amygdala (L), visual C (L, R), cerebellum, OFC (L), premotor area (L)
6	Carter et al. 2006	14	9/5	24.7	Delay Trace	fMRI	Delay: 50% Trace: 100%	Abstract coloured images	Shock	Online: SCR, US-expectancy rating. Post: CS-US-contingency rating	**Delay and Trace conditioning (assessed as correlation between BOLD and SCR):** amygdala (L), hippocampus (L, R), occipital C (Post pole). **Delay and Trace conditioning (assessed as correlation between BOLD and US-expectancy):** mid frontal G (L, R), parahippocampal G (L)
7	Cheng et al. 2003	20	8/12	24.85	Delay	fMRI	100%	Visual cue	Shock	Online: SCR	**Delay conditioning (assessed as (paired>unpaired subjects); ROI analysis):** amygdala (R), mid occipital G (R)
8	Cheng et al. 2006	17 13	8/9 4/9	23.35 22.38	Delay Trace	fMRI	100%	Coloured lights	Shock	Online: SCR, US-expectancy rating	**Delay and Trace conditioning (assessed as (CS+response trials > CS+ nonresponse trials); ROI analysis):** amygdala (R)
9	Cheng et al. 2007	12	6/6	20.4	Delay	fMRI	100%	Visual cue	Shock	Online: SCR, US-expectancy rating	**Delay Conditioning (assessed as (CS trials with early period CR > CS trials with late period CR); ROI analysis):** amygdala (R)
10	Cheng et al. 2008	11	6/5	23.6	Delay Trace	fMRI	100%	Tones	Air blast	Online: eye blink. Post: CS-US-contingency questionnaire	**Delay and Trace conditioning (assessed as (late acquisition>early acquisition); ROI analysis):** MTL (L, R). **Delay and Trace conditioning (assessed as (delay and trace)>baseline; ROI analysis):** Cerebellum (L). **Trace conditioning (assessed as (trace>delay); ROI analysis):** MTL (R)
11	Dimitrova et al. 2004	20	11/9	26.2	Delay	fMRI	100%	Tone	Shock	Online: heart rate, EMG (leg withdrawal reflex)	**Delay Conditioning (assessed as (Extinction – unpaired phase)):** inf temporal G (L), Hippocampus (R), med temporal G (R, L), fusiform G (R). **Delay conditioning (assessed as linear regression in the acquisition phase):** fusiform G (R), Hippocampus (R), inf temporal G (R), med temporal G (R), lingual G (R), sup temporal G (R)
12	Doronbekov et al. 2005	10	10/0	23.4	Delay	PET	?	Photos	Aversive videotape	Online: CS-fear rating	**Delay conditioning (assessed as (second photo phase > first photo phase)):** amygdala (R), PCC (L), sup frontal G (R), sup temporal G (L). **Delay conditioning (assessed as (conditioning >control condition)):** amygdala (R), PCC (L), parieto-occipital S (R)
13	Dunsmoor et al. 2007	18	7/11	30.17	Delay	fMRI	100% 50%	Tones	Noise	Online: SCR, US-expectancy rating	**Delay conditioning (with increasing CS-US-pairing-rate relative to baseline):** ACC (L, R), amygdala (L, R), fusiform G (L, R), inf occipital G (L), precentral G (L), precuneus (L). **Delay conditioning (with 50% CS-US-pairing relative to 100% and CS-):** DPFC (L), insula (L, R)
14	Dunsmoor et al. 2008	18	7/11	30.17	Delay	fMRI	100% 50%	Tones	Noise	Online: SCR, US-expectancy rating	**Delay conditioning (assessed (as CS50+ > CS100); Regions demonstrating UR diminution):** amygdala (R), ACC (L, R), auditory C (R), cerebellum (L, R), DLPFC (L), inf parietal Lo (L, R), thalamus (L, R)
15	Fischer et al. 2000	8	0/8	25.6	Delay	PET	25%	Neutral or aversive videotapes	Shock	Online: non-specific electrodermal fluctuations (NSF), SCL, state anxiety (STAI-S), subjective units of distress (SUD), US-expectancy rating	**Delay conditioning (assessed as (rCBF before > rCBF after paired shocks); Regions with increased rCBF):** ACC (L, R), cerebellum, PFC (R), hypothalamus (L, R), midbrain central gray, globus pallidus (L), thalamus (L, R). **Delay conditioning (assessed as (rCBF before > rCBF after paired shocks); Regions with decreased rCBF):** ACC (L), amygdala (L, R), OFC (L), PFC (L, R), occipital C (L, R), parietal C (L, R), temporal C (L, R). **Delay conditioning (assessed as (rCBF before > rCBF after unpaired shocks); Regions with increased rCBF):** ACC (R), PFC (R), hypothalamus (L, R), insula (L), midbrain central gray, putamen (L), thalamus (R). **Delay conditioning (assessed as (rCBF before > rCBF after unpaired shocks); Regions with decreased rCBF):** ACC (L), amygdala (R), cingulate C (L) (BA 26, 29, 30), OFC (L), hippocampus (R), occipital C (L, R), temporal C (L, R). **Delay conditioning (assessed as (paired x unpaired shocks)):** cerebellum (L), temporal C (R)
16	Fischer et al. 2002	6	0/6	27.8	Delay	PET	33%	Visual white noise; snake videotapes	Shock	Online: SCR	**Delay conditioning (biologically relevant CS; Regions with increased rCBF):** frontal C (R). **Delay conditioning (biologically relevant CS; Regions with decreased rCBF):** hippocampus (L), temporal C (L, R)
17	Fredrikson et al. 1995	16	0/16	31.4	Delay	PET	100%	snake and spider videotape	Shock	Online: heart rate, SCR, state anxiety (STAI-S), subjective units of distress (SUD)	**Delay conditioning (assessed as (scans before>after shock delivery); Regions with increased rCBF):** ACC (L), PCC (L), hypothalamus (L, R), parietal C (L), premotor area (L), SI (L), thalamus (L, R). **Delay conditioning (assessed as (scans before>after shock delivery); Regions with decreased rCBF):** secondary visual C (L).
18	Furmark et al. 1997	8	0/8	30.4	Delay	PET	100%	Snake video	Shock	Online: SCR	**Delay conditioning (assessed as (scans before>after shock delivery)):** amygdala (L)
19	Gottfried and Dolan 2004	16	7/9	24	Delay Extinction	fMRI	50%	Neutral faces	Odours	Online: RT (indication task). Post: CS-US-contingency interview, CS-valence ratings	**Delay conditioning (assessed as (CS+unpaired>CS-)):** dorsomedial amygdala (R), insula (L, R), rostromedial OFC (L), vent midbrain (L). **Delay conditioning + Extinction:** amygdala (L, R), rostromedial OFC (L), VMPFC (L), insula (R), vent striatum (L, R). **Extinction (assessed as (CS+unpaired>CS-)):** amygdala (L, R), caudomedial OFC (R), VMPFC (L), insula (L, R). **Extinction – Conditioning:** amygdala (L, R), cau OFC (R), med OFC (R)
20	Hugdahl et al. 1995	5	5/0	22	Delay	PET	100%	Tones	Shock	Pre: SCR. Post: SCR	**Delay conditioning (assessed as (Extinction – Habituation)):** DLPFC (R), inf frontal C (R), mid frontal C (L), OFC (R), sup frontal C (R), inf temporal C (R), mid temporal C (R), temporo-occipital junction (L),
21	Jensen et al. 2003	11	6/5	28	Delay	fMRI	33%	Geometric visual figures	Shock	-	**Delay conditioning (assessed as (CS+unpaired>CS-)):** ACC (R), ant insula (L, R), vent striatum (L, R)
22	Klucken et al. 2008	32	14/18	23.26	Delay	fMRI	100%	Geometrical visual figures	Aversive pictures (IAPS)	Online: SCR. Post: CS-valence, -arousal, -fear, -disgust ratings, CS-US-contingency rating	**Delay conditioning (assessed as (CS+>CS-)):** ACC (L, R), amygdala (R), insula (L), lat OFC (L), N accumbens (L, R), occipital C (L), thalamus (L)
23	Knight et al. 1999	10	4/6	27.4	Delay	fMRI	100%	Light	Shock	-	**Delay conditioning (assessed as (paired>control group); ROI analysis):** ACC, retrosplenial C, visual C
24	Knight et al. 2004	30	13/17	24.5	Delay Extinction	fMRI	100%	Light	Shock	Online: SCR	**Delay conditioning (assessed as (paired>control group); ROI analysis):** amygdala (L, R), hippocampus (L). **Extinction (assessed as (paired>control group); ROI analysis):** amygdala (L, R), hippocampus (L)
25	Knight et al. 2004a	17	8/9	23	Delay Trace	fMRI	100%	Geometric visual figure	Shock	Online: SCR, US-expectancy rating	**Delay and Trace conditioning (assessed as (CS+ delay and trace)>CS-; ROI analysis):** ACC, mid occipital G (L, R), supramarginal G (L), med thalamus (L, R). **Delay and Trace conditioning (Regions with decreased activation):** ACC, PCC, sup frontal G (L), hippocampus, inf temporal G (L), mid temporal G, sup temporal G (L), postcentral G (R). **Trace conditioning (assessed as trace interval > (CS+ and CS-)):** mid frontal G (L, R), hippocampus, frontal operculum (L, R), inf parietal L (R), SMA
26	Knight et al. 2005	9	4/5	28.33	Delay	fMRI	80%	Tones	Noise	Online: SCR. Online: SCR	**Delay conditioning (assessed as association with conditioned SCR):** amygdala (R), cerebellum (R), insula (R), med PFC (L), mid frontal G (L), precentral G (L), sup temporal G (L)
27	LaBar et al. 1998	10	5/5	22.5	Delay Extinction	fMRI	100%	Geometric visual figure	Shock	Post: CS-US-contingency rating. Follow-up study (same sample): SCR	**Delay conditioning (assessed as (CS+>CS-)):** ros ACC, cau ACC, mid frontal G (L), sup frontal G (L), periamygdaloid C (L), precentral G (R), striatum (L, R), sup temporal G (L). **Extinction (assessed as (CS+>CS-)):** amygdala (L), caudate N (L), mid frontal G (L), sup frontal G (L, R), precentral G (R), sup temporal G (R)
28	Li et al. 2008	12	4/8	-	Delay	fMRI	100%	Odour	Shock	Pre: discrimination test, US-intensity, -valence, - familiarity ratings. Online: SCR. Post: discrimination test, US-intensity, -valence, - familiarity ratings	**Delay conditioning (assessed as discrimination of perceptual cues; CS+>CS-; postconditioning > preconditioning):** amygdala, OFC (L, R), ant piriform C, post piriform C.
29	Logan and Grafton 1995	12	5/7	23	Delay	PET	90%	Tone	Air blast	Online: eye blink	**Delay conditioning (assessed as(paired > unpaired scans)):** inf cerebellum (L, R), ant cerebellar vermis, cerebellar C (L), cerebellar deep nuclei or pontine tegmentum (L), hippocampal formation (R), vent striatum (L, R), inf thalamus/red N (R), mid temporal G (R), occipitotemporal fissure (L)
30	Molchan et al. 1994	8	0/8	22.3	Delay Extinction	PET	100%	Tone	Air blast	Online: eye blink	**Delay conditioning (assessed as (paired > unpaired scans); Regions with increased rCBF:** PCC (L), transverse temporal C (L, R). **Delay conditioning (assessed as (paired > unpaired scans); Regions with decreased rCBF:** cerebellar C (R), inf frontal C (R), insula (R), neostriatum (R), inf parietal C (R). **Extinction (assessed as (unpaired > paired scans); Regions with increased rCBF:** inf frontal C (L, R). **Extinction (assessed as (unpaired > paired scans); Regions with decreased rCBF:** PCC (L), pons (L), sup temporal C (L, R)
31	Morris et al. 1997	6	6/0	32.7	Delay	PET	100%	Faces	Noise	Online: SCR. Post: CS-US-contingency-awareness assessment	**Delay conditioning (assessed as (CS+>CS-)):** OFC (R), sup frontal G (R), pulvinar N of the thalamus (R), anterolateral thalamus (R)
32	Morris et al. 1998	6	6/0	27.7	Delay	PET	40%	Tones	Noise	Online: SCR, discrimination task. Post: CS-US-contingency -awareness interview	**Delay conditioning (assessed as (CS+ > CS-)):** OFC (R). **Delay conditioning (assessed as auditory cortex regression analysis).** amygdala (L, R), OFC (R), basal forebrain, med geniculate N (L)
33	Morris and Dolan 2004	12	-	-	Delay	fMRI	33%	Neutral Faces	Noise	Online: RT (decision task), SCR (unusable)	**Delay conditioning (assessed as (CS+>CS- during acquisition)):** ACC (R), dor amygdala (L, R), insula (R), post thalamus (L, R).
34	Nitschke et al. 2006	21	10/11	19	Trace	fMRI	100%	Geometric visual figure	Aversive pictures (IAPS)	-	**Trace conditioning (assessed as (anticipation of aversive > neutral stimuli); ROI analysis):** dor ACC, ros ACC, amygdala (L, R) DLPFC (R), OFC (L, R), insula (L, R)
35	Petrovic et al. 2008	27	27/0	-	Delay	fMRI	50%	Faces	Shock	Pre: CS-sympathy ratings. Online: SCR. Post: CS-sympathy ratings, CS-US-contingency-awareness interview.	**Delay Conditioning (assessed as (CS+>CS-); ROI analysis):** amygdala (L, R), fusiform G (L, R)
36	Phelps et al. 2001	12	6/6	-	Delay	fMRI	0%	Blue and yellow squares	Shock	Online: SCR. Post: CS-US-contingency-awareness interview	**Delay conditioning (assessed as (threat vs. safe conditions)):** ACC, dor amygdala (L), basal forebrain, PFC, insula (L, R), PMA (R), striatum
37	Phelps et al. 2004	11	5/6	-	Delay Extinction	fMRI	33%	Geometric visual figure	Shock	Online: SCR	**Delay conditioning (assessed as (CS+>CS-)):** caudate N (L, R), dor ACC, insula (L, R), IPL (L, R). **Extinction (assessed as (CS+>CS-)):** caudate N (R), dor ACC, insula (L, R)
38	Pine et al. 2001	7	4/3	33.6	Delay	fMRI	100%	Coloured lights	Air blast	-	**Delay conditioning (assessed as (CS+>CS-);ROI analysis):** amygdala (R)
39	Ploghaus et al. 1999	12	7/5	26	Delay	fMRI	100%	Coloured lights	Thermal stimulus	Post: CS-US-contingency-awareness interview	**Delay conditioning (assessed as (anticipation of aversive > neutral stimuli)):** cerebellum, insula, MFL
40	Schiller et al. 2008	17	9/8	-	Delay	fMRI	33%	Mildly angry faces	Shock	Online: SCR	**Delay Conditioning (assessed as (CS+>CS-)):** dor ACC, amygdala (L), caudate N (L, R), sup frontal G, insula (L, R), midbrain (L), putamen (L), thalamus (R). **Reversal Delay Conditioning (assessed as (new CS->new CS+):** VMPFC
41	Schreurs et al. 1997	10	0/10	24.5	Delay Extinction	PET	100%	Tone	Air blast	Online: eye blink	**Delay conditioning (assessed as (paired > unpaired scans); Regions with increased rCBF):** lat temporooccipital G (L), sup temporal G (R), trans temporal G (R). **Delay conditioning (assessed as (paired > unpaired scans); Regions with decreased rCBF):** cerebellar C (L, R), inf prefrontal L (L), inf temporal pole (L), sup temporal pole (R). **Extinction (assessed as (unpaired > paired scans); Regions with increased rCBF):** cerebellar C (R). **Extinction (assessed as (unpaired > paired scans); Regions with decreased rCBF):** lat temporooccipital C (L), sup temporal C (R), trans temporal C (R)
42	Schreurs et al. 2001	10 1	0/10 0/11	22.3 69.2	Delay	PET	100%	Tone	Air blast	Online: eye blink. Post: CS-US-contingency-awareness interview	**Delay conditioning (assessed as paired scans; Regions with increased rCBF):** auditory C (L, R), PCC, MTL (L). **Delay conditioning (assessed as paired scans; Regions with decreased rCBF):** caudate N (R), cerebellum (L, R), inf PFC (L, R), midbrain
43	Straube et al. 2007	12	2/10	21.1	Delay	fMRI	50%	Visual stimulus	Shock	Pre: CS-valence, -arousal, -threat ratings. Online: RT (discrimination task, distraction task). Post: CS-valence, -arousal, -threat ratings. Follow-up study (independent sample): SCR, CS-valence ratings, US-intensity rating, CS-US-contingency rating	**Delay conditioning (assessed as (CS+unpaired>CS- )):** amygdala (L), brainstem (R), cingulate C (L, R), claustrum (R), DLPFC (L, R), DMPFC (L, R), insula (L, R), midbrain (R), PMA (L, R), SII (L, R), SMA (L, R), sup temporal S (L, R), thalamus (R).
44	Tabbert et al. 2005	18	6/12	-	Delay	fMRI	100%	Geometric visual figure	Shock	Online: SCR	**Delay conditioning (assessed as (CS+>CS-); ROI analysis):** amygdala (L), caudate N (L), OFC (L, R), occipital C (L), SMA (L)
45	Timmann et al. 1996	4	4/0	25.5	Delay	PET	100%	Tones	Shock	Online: eye blink, EMG (flexion response)	**Delay conditioning (assessed as correlation between rCBF and CR):** cerebellum, hippocampus (L, R), frontal C (L, R)
46	Yágüez et al. 2005	8	5/3	22	Delay Extinction	fMRI	Acquisition: 100% Anticipation: 50%	Coloured circles	Others, Air blast	-	**Delay conditioning (assessed as (CS->CS+ in the acquisition phase)):** ACC, cerebellum (L, R), mid ACC (R), inf frontal G (L, R), insula (L, R), postcentral G (L, R), SI (R), SII (L, R), SMA (L, R), sup temporal G (L, R). **Delay conditioning (assessed as (CS+>CS- in the anticipation phase)):** angular G (L, R), brainstem (R), mid ACC (R), cerebellum (L), DLPFC (R), inf frontal G (R), insula (L, R), SMA (R), supramarginal G (R). **Extinction (assessed as (CS+>CS-)):** ACC (R), mid ACC (R), DLPFC (R), mid frontal G (R), insula (L, R), SII (R), SMA (R)

Abbreviations: ACC: anterior cingulate cortex, ant: anterior; BA: Brodman area, cau: caudal, C: cortex, CR: conditioned response, CS: conditioned stimulus, dor: dorsal, DPFC: dorsal prefrontal cortex, DLPFC: dorsolateral prefrontal cortex, DMPFC: dorsomedial prefrontal cortex, EMG: electromyography, F: female, FOP: frontal operculum, G: gyrus, inf: inferior, IAPS: International Aversive Picture System, IPL: inferior parietal lobe, lat: lateral, L: left, Lo: lobule/lobe, M: male, med: medial, mid: middle, MFL: medial frontal lobe, MPFC: medial prefrontal cortex, MTL: medial temporal lobe, N: nucleus, No.: number, OFC: orbitofrontal cortex, post: posterior, PCC: posterior cingulate cortex, PFC: prefrontal cortex, PMA: premotor area, R: right, rCBF: regional cerebral blood flow, trans: transverse, RT: reaction time, ros: rostral, SCR: skin-conductance response, SCL: skin-conductance level, SI: primary somatosensory cortex, SII: secondary somatosensory cortex, SMA: supplementary motor area, S: sulcus; sup: superior, vent: ventral, VMPFC: ventromedial prefrontal Cortex, US: unconditioned stimulus.

### Data Analysis

The review provides a qualitative summary of neuroimaging findings on fear conditioning and extinction of the included empirical studies. These studies were classified according to the type of study design (delay, trace, and extinction), the modality of the CS and US, the contingency rate, and the independent assessment of the conditioned response. For each category, we extracted the absolute frequency of activated brain areas for the contrasts of interest. Moreover, we attempted to identify common and divergent activations across individual study results. Studies, reporting additional or different activation from those described in the core fear network, were examined for the following variables to shed light on reasons for the discrepant findings: conditioning design (delay, trace, and extinction), contingency rate, and characteristics of the CS and US. We refrained from statistically combining results from the studies due to the differences in their design.

## Results

### Included studies

**Figure 1:**
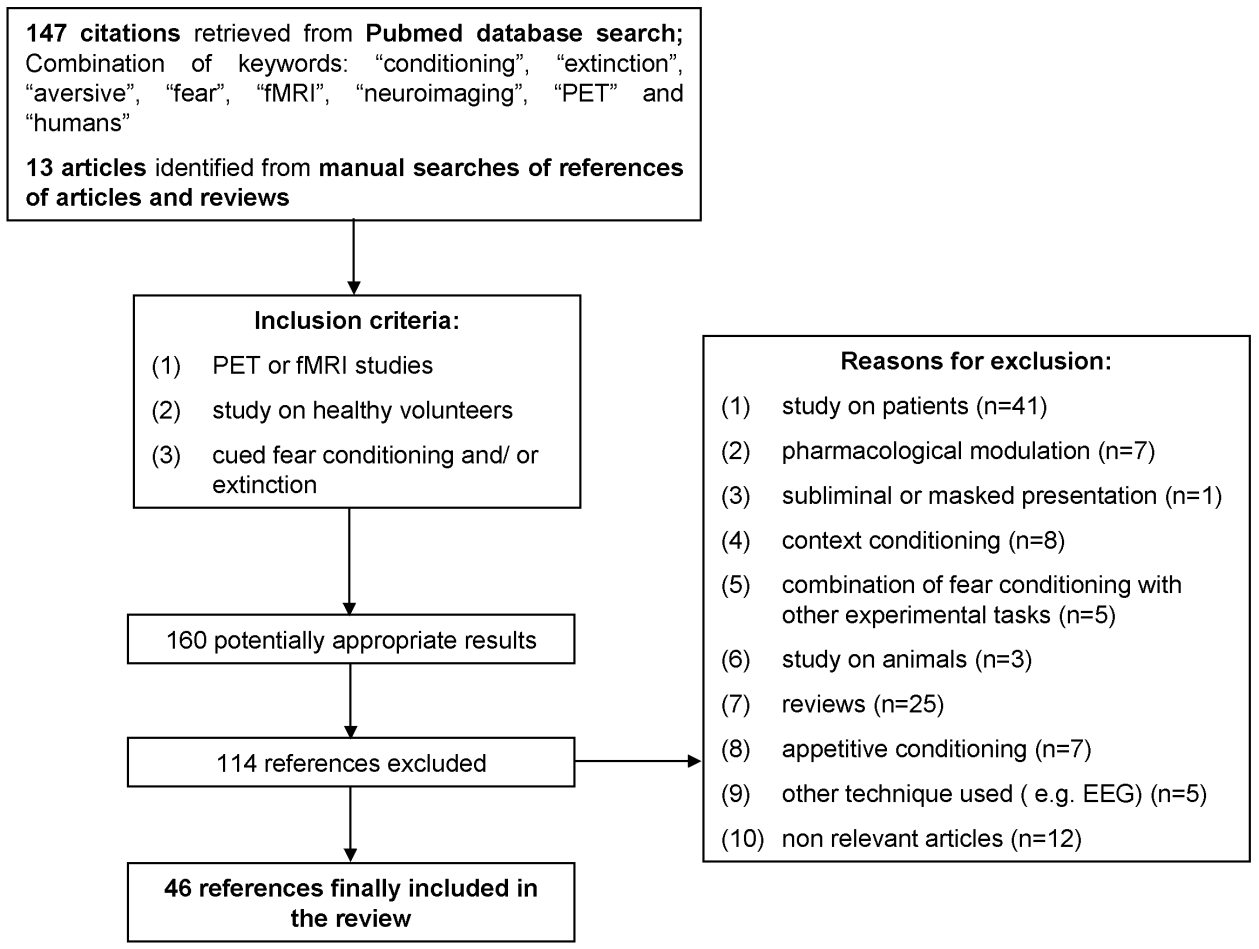
QUOROM flow chart used to identify studies for review.

Based on the literature search strategies, 147 citations were retrieved from the Pubmed database. Among these, we identified 33 relevant studies. Additionally, we examined the references of relevant articles and reviews. Thirteen citations met the selection criteria. As a whole, we reviewed 46 articles on human fear conditioning and/or extinction. Figure 1 shows the search and selection process. Forty studies exclusively used a delay conditioning paradigm during the acquisition phase (Table 1; No. 1–3, 5, 7, 9, 11–18, 20–23, 26, 28–29, 31–33, 35–36, 38–40, 42–45) [4], [5], [24]–[65]. Only two studies investigated solely trace conditioning during acquisition (Table 1; No. 4, 34) [66], [67], whereas four other studies used both delay and trace conditioning protocols (Table 1; No. 6, 8, 10, 25) [4], [5], [27], [28]. Extinction of learned fear was additionally reported by seven of the 40 delay conditioning studies (Table 1; No. 19, 24, 27, 30, 37, 41, 46) [37], [42], [44], [47], [51], [56], [60]. Thirty-two of the 46 studies are fMRI studies, 14 are PET studies. Table 1 contains information on empirical study characteristics and corresponding neuroimaging results.

### Summary of Findings

#### Brain regions involved in delay fear conditioning

**Figure 2:**
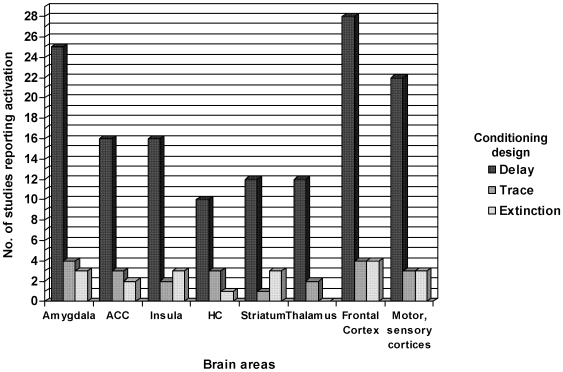
Brain areas involved in aversive conditioning and/or extinction. Different brain areas (with at least unilateral activation during aversive conditioning and/or extinction) are plotted against the x-axis. The number of studies out of 46 studies per brain region is plotted against the y-axis, taking into account the conditioning design which is delay conditioning in 40, trace conditioning in two, delay and trace conditioning in four, and extinction in seven studies.

As a major and stable result, the amygdala, the ACC and the insular cortex turned out to be crucial structures in the acquisition of aversive delay conditioning, independent of general design characteristics. Twenty-five of the 44 delay conditioning studies reported amygdala activation, with results varying with respect to the laterality of activation. While nine studies reported bilateral amygdala activation (e.g.[31], [49], [64]), eight studies detected left-lateralized (e.g. [27], [61], [65]), and eight right-lateralized activations (e.g.[53], [62]). Methodologically, nineteen of the 25 studies additionally tested for temporal interactions of amygdala activation or split up the acquisition phase into an early and late phase, in order to assess the temporal gradation in the signal intensity of the amygdala. Seventeen of these studies reported learning-related responses of the amygdala (e.g. [45], [48], [57], [66]): fourteen studies found initial increase and rapid decrease of activation during repeated exposure to unpleasant stimuli (e.g. [33], [44]), whereas three studies only reported increases of amygdala activation during the acquisition phase [40], [51], [58]. The remaining 19 delay conditioning studies did not report activation of the amygdala. Seventeen of them did not test for temporal aspects of amygdala activation (e.g. [39]). Sixteen delay conditioning studies found activation of the ACC (e.g. [25], [26]), five of the posterior cingulate cortex (PCC) (e.g. [30]), and two reported activation of the cingulate cortex [33], [57]. Sixteen studies detected insular activities (e.g [39], [54], [65]). These areas are all part of the classical key fear network as described previously [22], [23]. Activation of brain areas such as the hippocampus, the cerebellum, the thalamus, the striatum or the sensory cortices has been reported by fewer delay conditioning studies, underlining the considerable variability in neuroimaging findings. Hippocampal activity, mostly lateralized, was found for example by ten studies (e.g. [34]). Twelve studies showed activation of the striatum (including putamen, accumbens nucleus, caudate nucleus) (e.g. [39], [61]), whereas thalamic activity (including pulvinar, geniculate nucleus) was reported by twelve delay conditioning studies (e.g. [48]) (for details, see Figure 2). As argued below, we believe such differences in results to be methodological in origin [14].

#### Brain regions involved in trace fear conditioning

So far, only two fMRI studies have employed solely trace conditioning [66], [67] and four fMRI studies were conducted on both delay and trace conditioning [4], [5], [27], [28] (for details, see Table 1), all with either auditory, visual or tactile aversive stimulation. Again, the amygdala and the medial temporal lobe (MTL) were predominantly activated during the acquisition of trace conditioning in five studies (e.g. [4], [27], [66]). Activation of the ACC was apparent in three studies [5], [66], [67] and of the PCC in one study [5]. The hippocampus was bilaterally activated in three trace conditioning studies [5], [27], [66], and two studies showed additional activation of the insula [66], [67]. These fear-related structures such as the amygdala, the hippocampus, the ACC, the insula and the MTL were active independently of US-modality. Furthermore, activation was observed in different areas of the frontal cortex such as the dorsolateral prefrontal cortex (DLPFC) (e.g. [66]) or the middle frontal gyrus [5], [27] in four trace conditioning studies. Activation of other brain areas, such as the cerebellum, was reported in one study, of the motor cortices in three studies (e.g. [4], [5]) (for details, see Figure 2). Again, this variability in study results may be due to critical design characteristics, which will be discussed below.

#### Brain areas involved in fear extinction

Although extinction is very relevant in therapeutic settings, only seven studies with focus on extinction met criteria for our review [37], [42], [44], [47], [51], [56], [60]. All seven used a classical delay conditioning design during acquisition. Six studies used a tactile US (e.g. [51]), and one an olfactory US [37]. Three of the seven studies reported major activation foci in the amygdala [37], [42], [44], two in the ACC [51], [60], one study in the PCC [47] and three in the insula (e.g. [37], [47]), whereas four studies observed activation in frontal regions such as the prefrontal cortex (PFC), and the ventromedial prefrontal cortex (VMPFC) (e.g. [60]). Activation of the hippocampus was found in only one study [42] (for details, see Figure 2).

Although consensus exists that the amygdala again plays an important role in extinction, a closer look reveals that the details about amygdala activation vary. As with acquisition, four of the seven studies reported habituation of the amygdala response during extinction [37], [42], [44], [51]. To assess the temporal gradation in signal intensity of the amygdala, two of them split up the extinction phase into an early and late phase [44], [51], and one study tested for time×condition interaction [37]. Knight and co-workers (2004) reported an increase of right amygdala and a decrease of left amygdala activation during extinction, by t-test comparison [42]. Three other studies that did not analyze temporal activation patterns failed to find amygdala activation [47], [56], [60].

#### The influence of CS-US-contingency

Contingency describes the rate of pairing between the previously neutral CS+ and the aversive US, and therefore the predictability of the US in relation to the CS. In some cases, the CS is paired with the US on every trial (continuous pairing), whereas in other conditioning designs, CS and US are paired intermittently.

Contingency rates in neuroimaging studies cited here are quite heterogeneous. Twenty-five studies used 100% contingency (e.g. [29], [43], [44], [63]), two employed an 80% or a 90% pairing rate [41], [46], six included a 50% partial reinforcement procedure (e.g. [37]), and eight described lower contingencies of 40%, 33%, 25% or 0% (e.g. [33], [48], [51], [52], [65]). Three studies used 100% and 50% contingency rates during different phases of the experiment [31], [32], [60]. Another study employed a continuous pairing design during trace conditioning and a 50% pairing rate during delay conditioning [27]. One study did not report any contingency rates [30]. Results of the studies cited here indicate that activation of the amygdala seems to be independent of contingency rate: While thirteen studies employing continuous (100%) pairing, eight studies using 50% reinforcement and six studies with 0%, 25%, 33%, 40% and 80% all reported amygdala activation, (e.g. [26], [29], [37], [49], [66]), others with the same pairing rates did not (e.g. [25], [60]).

Awareness about this CS-US-contingency, mediated by conscious US-expectancies or by explicit instruction about the CS-US-contingency, also influences brain activation. Participants were explicitly informed about the CS-US pairing before the experiment in some studies (e.g. [33]), but not in others (e.g. [44]).

Finally, the choice of contingency rates is related to a problem specific to neuroimaging studies: the choice of contrasts between conditions. In a continuous pairing paradigm where the CS+ is always presented with the US, contrasts may be calculated between CS+ and CS- (e.g. [58]), between paired und unpaired subjects (e.g. [42]), or between conditioned and pseudo-conditioned phases - in which CS and US are not correlated in time (e.g. [25]). In a partial-reinforcement design, CS+ may be paired or unpaired with the US. Here, contrasts are mainly calculated between CS+unpaired and CS- (e.g. [26]).

#### Characteristics of the CS and US

Neuroimaging studies on fear conditioning have used different types of conditioned and unconditioned stimuli. Conditioned stimuli were presented visually, acoustically or olfactory. Thirty-one studies used a visual cue as CS: five studies used coloured lights (e.g. [28], [42], [53]), one study photographs [30], and four videotapes (e.g. [34], [35]). Seven studies, however, employed photographs of human faces (e.g. [24], [26], [37], [49], [64]), and 14 used geometrical figures (e.g. [51], [58]). Fourteen investigations used auditory conditioned stimuli (e.g. [31], [41], [46], [66]), whereas only one study employed odours [45]. Again, activation of the amygdala was independent of CS-modality: five studies with auditory CS (e.g. [66]), 21 using a visual CS (e.g. [57]) and one study which employed an olfactory CS [45] reported amygdala activation.

Unconditioned stimuli differ in modality (auditory, olfactory, tactile, and visual), in salience, as well as in unpleasantness, factors that may all influence the neurobiology of fear learning. Twenty-four studies used electric shocks (e.g. [5], [27]–[29], [33], [44]). The intensity of the shock is often assessed and adjusted to an individual level described as “unpleasant but not painful”, such that voltage varied from 40 V to 70 V between participants (e.g. [30], [37], [43], [44], [61], [68]). Electrical stimuli were administered to different areas, such as the wrist (e.g. [44], [51], [52]), shin (e.g. [58]), foot (e.g. [27]), or finger (e.g. [33], [39]). Further tactile stimulations, such as air blasts are reported in eight studies [4], [25], [46], [53], [56], [60], thermal stimulation with hot water in one study [54], and painful phasic esphageal distention in another study [60]. Nine studies cited here included auditory US, such as loud unpleasant tones [26], [66], or loud white noises (e.g. [31], [41], [48], [49]) at intensities of 95dB to 100dB, for 500–1000 ms. A verbal stimulus, a human scream, was presented as unconditioned stimulus in one study [24]. Another study used an olfactory unconditioned stimulus in human fear conditioning, such as “rotten eggs” and “sweaty socks” [37]. Finally, pictures (IAPS; International Affective Picture System [69]) or aversive videotapes were presented as aversive stimuli in three studies [30], [40], [67].

**Figure 3:**
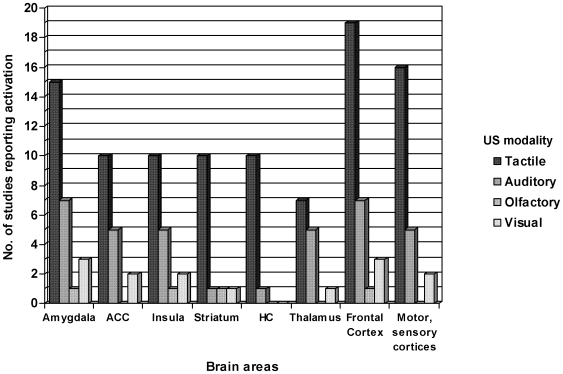
Brain areas involved in aversive conditioning according to the modality of the US. Different brain areas (with at least unilateral activation during aversive conditioning) are plotted against the x-axis. The number of studies out of 46 studies per brain region is plotted against the y-axis, taking into account US modality, which is tactile in 33 studies (such as electrical shocks), auditory in nine studies (such as noise), olfactory in one study (such as odors), or visual in three studies (such as aversive pictures).

Again, activation of the fear network was observed to be independent of US-modality. In spite of different USs, activations of the amygdala, ACC and insula were reported for every stimulus type. Of the 33 studies with tactile stimulation, fifteen found activation of the amygdala (e.g. [29]), ten of the ACC (e.g. [35]), and ten of the insular cortex (e.g. [39]). Other main activation foci for tactile stimuli concern the thalamus in seven (e.g. [46]), and the striatum in ten studies (e.g. [52]). Other regions such as the occipital cortex, motor or somatosensory cortices are also activated during tactile conditioning in 16 studies (e.g. [27], [35]). By contrast, the nine studies on auditory fear conditioning mainly report activation of the fear network, with emphasis on amygdala in seven (e.g. [31]), on ACC in five (e.g. [48]), and on insula in five studies (e.g. [41]). Moreover, activations of the motor or sensory cortices (e.g. auditory, occipital) are also apparent in five studies (e.g. [26]). The one study on olfactory conditioning mainly reports activations in amygdala, insula and orbitofrontal cortex (OFC) [37], areas that are also associated with the perception of disgust [70], [71]. All three studies on visual aversive conditioning reported activation of key fear areas such as the amygdala and ACC or the PCC [30], [40], [67]. Activation of the insula was found in two of the studies (e.g. [67]). Furthermore, activations of the DLPFC, OFC, thalamus, nucleus accumbens and the occipital cortex are apparent in these visual conditioning studies (e.g. [40]) (for details, see Figure 3).

Our review reveals that 38 of the reviewed studies employed different modalities of US and CS. Only five studies chose an auditory CS paired with an auditory US (e.g. [31], [41], [66]), and three were conducted on visual CS and US (e.g. [40]). Again, research is needed to quantify this effect of common CS-US-modality on neuroimaging results.

#### Independent assessment of the conditioning process

**Figure 4:**
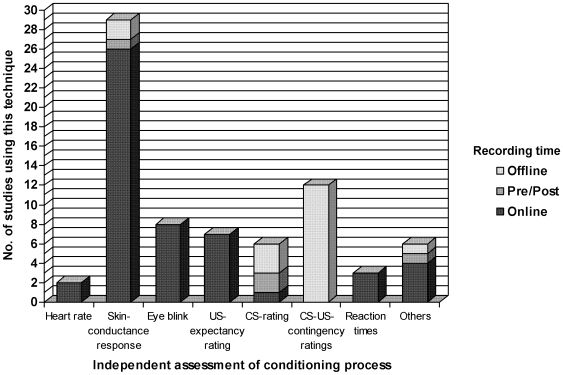
Number of studies employing an independent assessment of the conditioning process. Different independent assessments of the conditioning process which may be autonomous (such as skin-conductance responses), or behavioral (such as verbal ratings), are plotted against the x-axis. The number of studies out of 46 studies per technique is plotted against the y-axis taking into account if the technique is applied online during scanning, offline after scanning or offline before and after scanning (pre/post).

A control procedure to ensure that a physiological response towards the CS+ has actually occurred, with data from dependent variables other than brain activation, was used in 41 of the 46 studies cited here. Autonomous, endocrine, or behavioral responses, such as skin-conductance responses, heart rate, verbal responses (ratings of the CS, US-expectancy ratings, or CS-US-contingency assessment), reaction times, or eye-blink reflex qualify as parameters of successful conditioning. The majority of the studies employed independent measures online during scanning. Autonomous measures, such as heart rate, were applied in two [35], [63], skin-conductance responses in 26 (e.g. [4], [32], [50], [62], [64], [65]), and eye-blink startle response in eight studies (e.g. [24], [25], [46], [56]). Only three studies used SCR outside the scanner: before and after conditioning [38] or in an additional experiment [44], [57]. Online assessments of verbal responses, such as CS-ratings, were used in one study [30], and US-expectancy ratings in seven studies (e.g. [28], [31], [62]). Two studies compared ratings of the CS before and after scanning [57], [64]. Twelve studies employed CS-US-contingency ratings and three studies CS-ratings post experimentally (e.g. [27], [40]). To conclude, twenty-three studies combined different measurements of the conditioned response (e.g. [27], [44]) (for details, see Table 1, Figure 4). To summarize, objective measurements are necessary when studying conditioning, to verify that conditioned learning has indeed occurred.

## Discussion

This review deals with the neural correlates of human fear conditioning in current fMRI and PET studies. Our analysis indicates that neuroimaging studies on human fear conditioning and extinction activate a common core fear network which is in accordance with evidence from other sources (e.g. [17]). Some neuroimaging studies do not find these activations. This heterogeneity is not surprising taking into account the large methodological variety in imaging and design parameters. Methodological differences were found a) in the conditioning protocol (delay, trace), b) in the contingency rate (100%, 80%, 50% or less) and awareness, c) in the modality of CS and US (tactile, auditory, visual, olfactory), and d) with respect to the further assessment of the conditioned response (e.g. psycho-physiological measurements, verbal ratings).

Neuroimaging studies have substantially extended our understanding of fear conditioning and extinction, adding in vivo evidence from humans to previous electrophysiological and lesion studies from animals [3], [72]. Consistent with comparative animal data, neuroimaging investigations have corroborated the finding of a neural fear network activated during fear conditioning. Within this core fear network, key structures for the acquisition and the extinction of conditioned fear have been identified, although there is considerable methodological heterogeneity between studies, with some of them not reporting these activations. Furthermore, it turned out that anatomical regions relevant in fear conditioning are also involved in the extinction of fear memories. In conformity with animal and lesion data, our review indicates that the amygdala, as one principal structure of the limbic system, is one of the key regions involved in fear conditioning and extinction. Amygdala activation occurs in response to emotional stimuli and is therefore regarded as the gate keeper funnelling emotionally relevant information into different processing channels. This region is activated during conditioned-fear acquisition as well as during the expression of learned fear (see for an overview [23]). Furthermore, amygdala activation undergoes rapid habituation during acquisition and extinction that should be taken into account in neuroimaging studies (e.g. [26]). This typical response profile of the amygdala may not be detected by categorical comparisons of e.g. CS+ and CS-, as this contrast reflects time-invariant neural responses. Consequently, some studies carried out an analysis that tested for this type of time-dependent response profile. They set up a statistical model that allows characterizing the activation of the amygdala by a time by condition interaction. Therefore, we suppose that testing for interactions between conditions and time may reveal conditioning results that otherwise remain hidden, such as amygdala activation.

Furthermore, some brain regions, especially the MTL, are difficult to assess using echo-planar imaging (EPI) because they are highly vulnerable to susceptibility artifacts [73]. These differences may cause image distortion and signal dropout [73]–[75]. This might be another reason why some studies did not find amygdala activation during conditioning. Activation of the insula, another central structure for emotion processing, was also shown in 40% of the neuroimaging studies. Phelps and co-workers (2001) assume that the insula cortex conveys a cortical representation of fear to the amygdala [52], and that uncertainty about the advent of the aversive stimulus during intermittent pairing is reflected by insula and dorsal prefrontal cortex activation [31], [32], [76]. Another region belonging to the core fear conditioning and extinction network described by the majority of the cited neuroimaging studies is the ACC (for an overview, see [17], [26]). The ACC plays an important role in approach and avoidance learning [77] as well as in fear learning [78]. The frontal cortex is particularly crucial for emotional regulation and therefore for the extinction of conditioned fear. Although extinction is the essential process in therapeutic settings, only seven studies have so far focused on extinction. From both animal data and theoretical considerations, it is evident that fear extinction involves mainly interactions between cortical and subcortical structures, such as the PFC and the amygdala or the hippocampus (see for an overview [79]). As one of the principal structures of the brain's extinction circuitry, the PFC regulates the expression of fear by inhibiting the amygdala, such that the fear-conditioned stimulus is prevented from causing a conditioned fear response [79]–[81]. In this review, only one study reported hippocampal activation during extinction. This is surprising, because from other studies is known that the hippocampus and the VMPFC seem specifically important during late phases of extinction, and therefore for the retention of extinction [51], [82].

There are, however, considerable variances and discrepancies between studies. Whereas some studies only report activation of the core network, others do not find these activations or observe activation within additional brain regions, such as the hippocampus, striatum, sensory cortices or thalamus. The choice of conditioning protocol, CS-US contingency, and modality of the US seem to be very important factors modifying brain-activation patterns in fear conditioning studies.

Our review indicates that of these factors, the conditioning protocol has great impact on brain activation. Delay conditioning leads to more rapid learning of the CS-US association than trace conditioning [83]–[85]. Thus, from the experimental point of view, delay conditioning has the advantage of a shorter acquisition time, fewer trials, and a more rapid conditioning process than trace conditioning. Additionally, delay conditioning designs are known to extinguish associations faster than those established during trace conditioning [86]. Therefore, all studies that investigated extinction employed delay conditioning in advance. By contrast, in trace conditioning, CS is separated from the US by a temporal gap, resulting in prolonged acquisition times and a larger number of trials being required to form an association. The length of the temporal gap and its distance to the subsequent stimulus also exerts a strong influence. When the US is followed immediately by the next CS, *backward conditioning* (US-CS associations) or *contextual conditioning* can occur. In backward conditioning, the US is associated with the next CS, so that no conditioned response is established [87]. Contextual conditioning describes the association of the CS with contextual cues [88], [89]. Hence, there is no contiguity in trace conditioning. While in general, delay and trace conditioning involve comparable fear-related networks, activation of the hippocampus is typical of trace conditioning. In trace conditioning, hippocampal activation is required to bridge the gap between CS and US, retaining a memory trace which is needed to form an association between CS and US [90]. The hippocampus is involved in trace conditioning irrespective of the length of trace interval. However, animal data show that some neurons in the hippocampus encode the duration of trace interval [91]. Thus we assume that the level of hippocampal activation may be enhanced by increasing the length of trace interval.

Another important variable contributing to heterogeneity of neuroimaging results is the CS-US pairing or contingency rate. Effects of CS-US-contingency on conditioning have been repeatedly described in the psychological and behavioral literature [31], [32], [92]–[94]. Contingency rates determine how fast conditioned responses are acquired, and regulate extinction processes. Our review reveals that the activation of the core fear network consisting of amygdala, insula and ACC is independent of pairing rate, but the time courses of neural responses and the degree of activation may be influenced by contingency. In general, a predictable US is less aversive than an unpredictable US. Therefore, the continuous (100%) pairing of CS+ and US reduces fear responses and activity in fear-related brain areas [31], [32], and promotes the habituation of the amygdala [26], [44], [57], [58], relative to intermittent pairing. Nevertheless, the majority of the studies cited in this review employed a continuous pairing paradigm. In intermittent procedures, US expectancy and response frequency is decreased, which slows conditioning and prolongs the extinction phase [31], [32], [51]. The choice of these pairing parameters has important implications for analysis of imaging data. First, in the light of habituation processes, analysis of time by condition interactions may well improve the detection of amygdala activation. Second, the choice of the contingency rate influences the definition of contrasts of interest between test and control conditions. The cited studies differ in their contrasts of interest which may also influence resulting activation and complicate comparing studies even further. For example, in a 100% pairing design resulting differences in neural responses may be confounded by US-induced BOLD changes. In contrast, in a partial reinforcement design, differences in neural responses are only due to the anticipation of the US.

Our review also illustrates that there is an ongoing controversy on the role of contingency awareness. It seems clear that awareness of the CS-US contingency bridges the CS-US gap in trace conditioning [95], [96]. Therefore, it may be very important for trace conditioning, but less so for delay conditioning. Still, this topic requires further investigation. While some researchers found autonomic fear reactions only in contingency-aware subjects, others reported activation of the fear-network independently of contingency awareness [40], [97], [98]. For example, Phelps et al. (2001) showed that instructions alone can induce fear and that activation of the amygdala can occur without direct experience of the aversive event [52]. Tabbert et al. (2006) explicitly investigated the effect of contingency awareness. They either informed their subjects about the relationship of CS and US or prevented contingency detection by employing a distracter figure or a working-memory task. Amygdala and the OFC were only activated in the unaware group [98], but Klucken et al. (2008) found activation of fear-related areas independent of awareness [40]. However, robust conditioned skin-conductance responses have been observed only in aware participants who acquired a cognitive representation of CS-US-contingencies, and who were able to recall the correct contingency [97]. At this moment, concrete advice as to whether participants should be informed about contingency to obtain faster conditioning responses, is premature.

Concerning the modality of the US and CS, 33 of the 46 studies employed a tactile US, making it the most frequently applied US. Only nine studies used auditory aversive stimuli which may be due to the surrounding and interfering scanner noise. To the best of our knowledge, the problem of scanner noise as being aversive itself has not been discussed so far. The activation of the key fear network including amygdala, ACC and insula seems to be independent of the applied stimuli (auditory, olfactory, tactile, and visual). Nevertheless, many studies do not show activation of the key fear network or observe modality-specific activations. In fear conditioning with tactile US, activation of the thalamus, the striatum, somatosensory and of motor cortices is often reported. These areas are also associated with the nociceptive system, pain anticipation and perception (e.g. [99]–[102]). The nociceptive system includes the somatosensory cortices, ACC, insula, prefrontal and parietal cortices [103]. Koyama et al. (2005) showed that ACC activation increases with the magnitude of expected pain, and pain-intensity [104]. The thalamus, a major relay site for nociceptive inputs to cortical and subcortical structures, is thought to be responsible for the onset plasticity in the amygdala during fear conditioning [105]. Therefore, we suggest that a “pain-fear network” may be activated during tactile fear conditioning. The one study on olfactory conditioning reported mainly activations of amygdala, ACC and OFC [37]. Odour perception is more often related to disgust than to fear. Disgust and fear are basic emotions with different elicitors and expressions, and appear to be mediated by different neuronal circuits [70], [71], [106], [107]. Therefore, further research is needed to clarify if olfactory conditioning activates a “disgust-fear-network” rather than a mere “fear-network”. To conclude, it seems likely that odours, visual or acoustic stimuli may weaken conditioning effects and may cause activations in different brain regions than electrical stimuli. But to the best of our knowledge, this has never been tested directly in neuroimaging studies. Again, research is needed to quantify the effect of common CS-US-modality on neuroimaging results.

Concerning the modality of the CS, the majority of the studies used visual stimuli as CS, especially photographs of human faces. Faces as CS might be more emotionally relevant to human subjects than tones or coloured lights [108]. However, there seems to be a gender-related effect that needs to be considered in neuroimaging studies. For example, in women, the presentation of faces leads to stronger and persisting amygdala activation, while amygdala activation in men decreases rapidly [109]. Moreover, it is known that the amount of preexposure influences the outcome of aversive learning. These phenomena, so called “*latent inhibition*” and “*US-preexposure effect*”, emphasize that novel and unknown CS and US produce more robust conditioning effects than familiar stimuli [31], [110]. The disadvantage of unfamiliar stimuli is the mixing of novelty effects and conditioning effects.

Finally, it is very important to ensure that conditioning really takes place by sampling a second psycho-physiological or behavioral measure to avoid contamination of successful conditioning with unsuccessful trials. Skin-conductance responses as measures of autonomic responses have been widely investigated and are well validated [41]. Classifying subjects as “responders” and “non-responders”, or classifying single trials as “successful” or “not successful” conditioning based on autonomous measures has proven extremely useful, to exclude erroneous trials or subjects from further analysis (e.g. [28], [51]). However, technical issues in the scanner environment have to be solved. Measurement of skin-conductance responses may well prolong the experiment beyond critical time values for such experimental designs. On the other hand, verbal ratings may easily be influenced and consciously manipulated. Alternatives are the assessment of heart rate, or of the startle reflex, which is an elegant measure if an eye-tracker or electromyography is available. In all, the combination of different psycho-physiological and behavioral methods has proven valuable to assure that conditioning has really taken place.

### Strengths and Limitations

To the best of our knowledge, this review is the first summarizing current literature on neuroimaging fear conditioning and extinction and providing an overview on similarities and heterogeneities between study results. In this review, we focused on discussing experimental factors that are typical for conditioning paradigms, such as the design (delay, trace), the contingency rate, the contrasts of interests, or the stimuli (CS, US), and that may contribute to the reported heterogeneity in neuroimaging results. Other experimental factors that may influence fear conditioning and fMRI-studies are, for example, the MR-sequence (e.g. [74], [75]), the sample size, gender of participants (e.g. [111], [112]), genetic variables (e.g. [17], [113]–[115]), or personality factors (e.g. [116]–[124]). These variables may also contribute to the diversity of neuroimaging results. Another limitation is that our search did not include conditioning studies that were conducted on context conditioning, on patients, on pharmacological interventions, or that included another experimental task. However, we excluded these studies to limit the number of potential influencing variables.

### Conclusion

This review provides an overview of 46 current neuroimaging studies on fear conditioning and extinction. Neuroimaging yields new in-vivo evidence with respect to humans revealing and corroborating a consistent pattern of key areas in aversive conditioning and extinction. These structures encompass the amygdala, ACC, and insular cortex for both associative conditioning and extinction. This confirms previous electrophysiological or lesion studies on animals. The key fear-related brain areas, such as amygdala, ACC and insula, are activated independently of specific design parameters. However, some studies still do not report these findings or observe additional modality-specific activations. We pinpointed a number of methodological differences between the functional imaging studies and conclude that these may contribute to the observed variance between results. Prime candidate factors for modifying brain activation patterns are the choice of conditioning protocol, CS-US contingency, and modality of the US. Thus, the contingency and timing parameters, the modality of the CS and US, as well as the assessment of conditioned responses are important for conducting and interpreting neuroimaging studies on fear conditioning and extinction.
